# (Nitrato-κ*O*)(2,2′:6′,2′′-terpyridine-κ^3^ *N*,*N*′,*N*′′)palladium(II) nitrate

**DOI:** 10.1107/S2414314621000857

**Published:** 2021-01-29

**Authors:** Kwang Ha

**Affiliations:** a Chonnam National University, School of Chemical Engineering, Research Institute of Catalysis, Gwangju, Republic of Korea; Sunway University, Malaysia

**Keywords:** crystal structure, palladium(II) complex, square-planar structure, 2,2′:6′,2′′-terpyridine, tridentate ligand, nitrate salt

## Abstract

The title complex, [Pd(NO_3_)(C_15_H_11_N_3_)]NO_3_, comprises a cationic Pd^II^ complex and a nitrate anion. In the complex, the Pd^II^ cation is four-coordinated in a distorted square-planar coordination geometry defined by the three N atoms of the tridentate 2,2′:6′,2′′-terpyridine ligand and one O atom from the NO_3_^−^ anion. In the crystal, the complex mol­ecules are stacked in columns along the *a* axis being connected by π–π stacking [closest inter-centroid separation between pyridyl rings = 3.878 (3) Å]. The connections between columns and anions to sustain a three-dimensional architecture are C—H⋯O hydrogen bonds.

## Abstract 2

The central Pd^II^ ion of the complex cation has an N_3_O square-planar coordination sphere defined by the three N atoms of the tridentate 2,2′:6′,2′′-terpyridine ligand and one O atom from the NO_3_^−^ anion.

## Graphical abstract

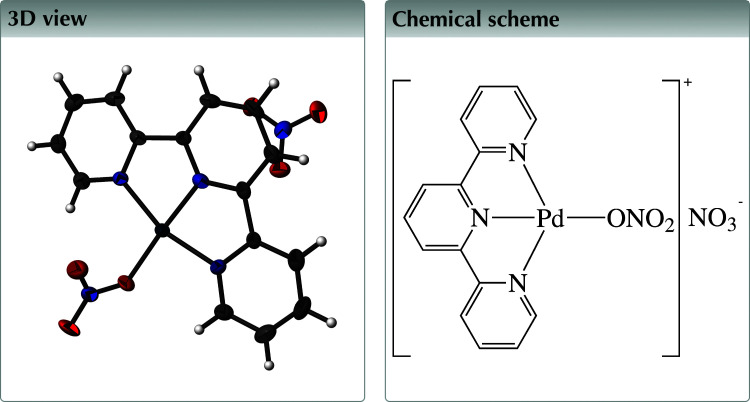

## Structure description

With reference to the title complex, [Pd(terpy)(NO_3_)](NO_3_) (terpy = 2,2′:6′,26′6′-terpyridine), the crystal structures of related Pd^II^ complexes [Pd(terpy)(pyridine)](ClO_4_)_2_ (Bugarčić *et al.*, 2004 ▸), [Pd(terpy)(NO_3_)](NTf_2_) [NTf_2_ = bis­(tri­fluoro­methyl­sulfon­yl)amide anion; Illner *et al.*, 2009 ▸) and [Pd_2_(terpy)_2_(NO_3_)]_2_(PF_6_)_6_·CH_3_CN (Mei *et al.*, 2007 ▸) have been determined previously.

**Figure 1:**
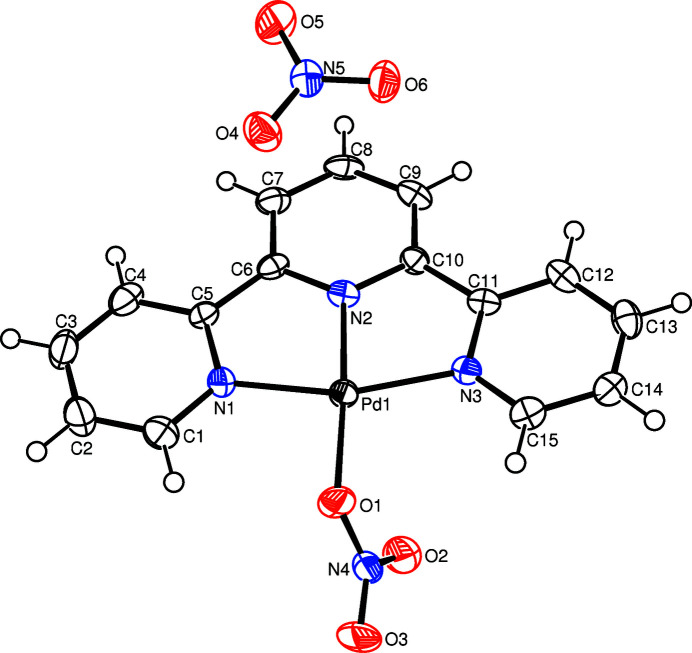
The mol­ecular structure of the title complex showing the atom labelling and displacement ellipsoids drawn at the 50% probability level for non-H atoms.

**Figure 2:**
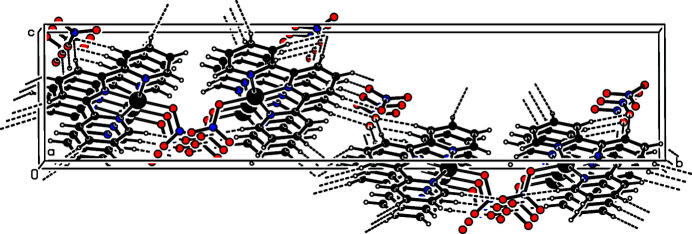
A view of the packing in the crystal of the title complex, viewed approximately along the *a* axis. Hydrogen-bonding inter­actions are drawn as dashed lines.

The title complex comprises a cationic Pd^II^ complex [Pd(terpy)(NO_3_)]^+^ and an NO_3_^−^ anion (Fig. 1 ▸). In the complex, the central Pd^II^ cation is four-coordinated in a distorted square-planar coordination geometry defined by the pyridyl N1, N2 and N3 atoms derived from the tridentate terpy ligand and the O1 atom from the nitrato ligand. The tight N—Pd—N chelating angles of <N1—Pd1—N2 = 81.26 (17)° and <N2—Pd1—N8 = 81.03 (16)° contribute to the distortion of the square-plane. The Pd—N [1.917 (4) to 2.030 (4) Å] and Pd—O [2.028 (3) Å] bond lengths are close. The pyridine rings of the terpy ligand are located approximately parallel to the least-squares plane of the PdN_3_O unit [maximum deviation = 0.023 (2) Å], with dihedral angles of 1.4 (2)° (ring N1/C1–C5), 3.1 (2)° (ring N2/C6–C10) and 3.0 (2)° (ring N3/C11–C15). In the crystal (Fig. 2 ▸), the complex mol­ecules are stacked in columns along the *a* axis. Within the columns, numerous inter­molecular π–π inter­actions between adjacent pyridine rings are present. For *Cg*1 (the centroid of ring N2/C6–C10) and *Cg*2^i^ [the centroid of ring N3/C11–C15; symmetry code: (i) *x* + 1, *y*, *z*], the centroid-centroid distance is 3.878 (3) Å and the dihedral angle between the ring planes is 3.2 (3)° (Spek, 2020 ▸). The complex cations and anions form inter­molecular C—H⋯O hydrogen bonds (Table 1 ▸) to stabilize the three-dimensional packing.

## Synthesis and crystallization

To a solution of Pd(NO_3_)_2_·2H_2_O (0.1320 g, 0.495 mmol) in acetone (30 ml) was added 2,2′:6′,2′′-terpyridine (0.1179 g, 0.505 mmol) followed by stirring for 3 h at room temperature. The formed precipitate was separated by filtration, washed with acetone and dried at 323 K to give a light-yellow powder (0.2123 g). Yellow crystals of the product suitable for X-ray analysis were obtained by slow evaporation of its CH_3_NO_2_ solution at room temperature.

## Refinement

Crystal data, data collection and structure refinement details are summarized in Table 2 ▸.

## Supplementary Material

Crystal structure: contains datablock(s) I. DOI: 10.1107/S2414314621000857/tk4067sup1.cif

Structure factors: contains datablock(s) I. DOI: 10.1107/S2414314621000857/tk4067Isup2.hkl

CCDC reference: 2058389

Additional supporting information:  crystallographic information; 3D view; checkCIF report

## Figures and Tables

**Table 1:** Hydrogen-bond geometry (Å, °)

*D*—H⋯*A*	*D*—H	H⋯*A*	*D*⋯*A*	*D*—H⋯*A*
C3—H3⋯O2^i^	0.94	2.55	3.419 (7)	153
C4—H4⋯O6^ii^	0.94	2.37	3.303 (7)	172
C7—H7⋯O6^ii^	0.94	2.30	3.231 (6)	171
C8—H8⋯O5^iii^	0.94	2.43	3.088 (6)	127
C9—H9⋯O6^iv^	0.94	2.35	3.254 (6)	160
C13—H13⋯O5^v^	0.94	2.46	3.402 (7)	176
C15—H15⋯O3^vi^	0.94	2.38	3.280 (7)	161

Symmetry codes: (i) 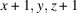 ; (ii) 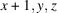 ; (iii) 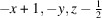 ; (iv) 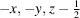 ; (v) 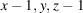 ; (vi) 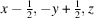 .

**Table 2:** Experimental details

Crystal data	
Chemical formula	[Pd(NO_3_)(C_15_H_11_N_3_)]NO_3_
*M*_r_	463.69
Crystal system, space group	Orthorhombic, *P* *n* *a*2_1_
Temperature (K)	223
*a*, *b*, *c* (Å)	6.2190 (2), 33.9728 (15), 7.4819 (3)
*V* (Å^3^)	1580.75 (11)
*Z*	4
Radiation type	Mo *K*α
μ (mm^−1^)	1.22
Crystal size (mm)	0.21 × 0.14 × 0.06

Data collection	
Diffractometer	PHOTON 100 CMOS detector
Absorption correction	Multi-scan (*SADABS*; Bruker, 2016 ▸)
*T*_min_, *T*_max_	0.688, 0.745
No. of measured, independent and observed [*I* > 2σ(*I*)] reflections	41749, 3116, 2745
*R*_int_	0.084
(sin θ/λ)_max_ (Å^−1^)	0.618

Refinement	
*R*[*F* ^2^ > 2σ(*F* ^2^)], *wR*(*F* ^2^), *S*	0.027, 0.048, 1.09
No. of reflections	3116
No. of parameters	244
No. of restraints	1
H-atom treatment	H-atom parameters constrained
Δρ_max_, Δρ_min_ (e Å^−3^)	0.34, −0.43
Absolute structure	Flack *x* determined using 1141 quotients [(*I* ^+^)−(*I* ^−^)]/[(*I* ^+^)+(*I* ^−^)] Parsons *et al.* (2013 ▸).
Absolute structure parameter	0.006 (16)

Computer programs: *APEX2* and *SAINT* (Bruker, 2016 ▸), *SHELXT2014/7* (Sheldrick, 2015*a* ▸), *SHELXL2014/7* (Sheldrick, 2015*b* ▸) and *ORTEP-3 for Windows* (Farrugia, 2012 ▸).

## full crystallographic data

### Crystal data

**Table:**

[Pd(C_15_H_11_N_3_)(NO_3_)]NO_3_	*D*_x_ = 1.948 Mg m^−^^3^
*M**_r_* = 463.69	Mo *K*α radiation, λ = 0.71073 Å
Orthorhombic, *P**n**a*2_1_	Cell parameters from 9942 reflections
*a* = 6.2190 (2) Å	θ = 2.4–27.7°
*b* = 33.9728 (15) Å	µ = 1.22 mm^−^^1^
*c* = 7.4819 (3) Å	*T* = 223 K
*V* = 1580.75 (11) Å^3^	Plate, yellow
*Z* = 4	0.21 × 0.14 × 0.06 mm
*F*(000) = 920	

### Data collection

**Table:**

PHOTON 100 CMOS detector diffractometer	2745 reflections with *I* > 2σ(*I*)
Radiation source: sealed tube	*R*_int_ = 0.084
φ and ω scans	θ_max_ = 26.1°, θ_min_ = 2.4°
Absorption correction: multi-scan (SADABS; Bruker, 2016)	*h* = −7→7
*T*_min_ = 0.688, *T*_max_ = 0.745	*k* = −41→42
41749 measured reflections	*l* = −9→9
3116 independent reflections	

### Refinement

**Table:**

Refinement on *F*^2^	Secondary atom site location: difference Fourier map
Least-squares matrix: full	Hydrogen site location: inferred from neighbouring sites
*R*[*F*^2^ > 2σ(*F*^2^)] = 0.027	H-atom parameters constrained
*wR*(*F*^2^) = 0.048	*w* = 1/[σ^2^(*F*_o_^2^) + (0.0152*P*)^2^ + 0.8349*P*] where *P* = (*F*_o_^2^ + 2*F*_c_^2^)/3
*S* = 1.09	(Δ/σ)_max_ = 0.001
3116 reflections	Δρ_max_ = 0.34 e Å^−^^3^
244 parameters	Δρ_min_ = −0.43 e Å^−^^3^
1 restraint	Absolute structure: Flack *x* determined using 1141 quotients [(*I*^+^)-(*I*^-^)]/[(*I*^+^)+(*I*^-^)] Parsons *et al.* (2013).
Primary atom site location: structure-invariant direct methods	Absolute structure parameter: 0.006 (16)

### Special details

**Table:**

Geometry. All esds (except the esd in the dihedral angle between two l.s. planes) are estimated using the full covariance matrix. The cell esds are taken into account individually in the estimation of esds in distances, angles and torsion angles; correlations between esds in cell parameters are only used when they are defined by crystal symmetry. An approximate (isotropic) treatment of cell esds is used for estimating esds involving l.s. planes.
Refinement. Hydrogen atoms on C atoms were positioned geometrically and allowed to ride on their respective parent atoms: C—H = 0.94 Å and *U*_iso_(H) = 1.2*U*_eq_(C). The Flack parameter = 0.006 (16) after the final cycles of refinement.

### Fractional atomic coordinates and isotropic or equivalent isotropic displacement parameters (Å^2^)

**Table:**

	*x*	*y*	*z*	*U*_iso_*/*U*_eq_	
Pd1	0.22807 (4)	0.15784 (2)	0.48682 (9)	0.02233 (10)	
O1	0.1702 (6)	0.21529 (10)	0.4313 (4)	0.0343 (10)	
O2	0.2291 (7)	0.19950 (12)	0.1521 (6)	0.0463 (10)	
O3	0.1758 (7)	0.26017 (11)	0.2256 (6)	0.0540 (13)	
N1	0.4977 (6)	0.16769 (12)	0.6321 (6)	0.0261 (10)	
N2	0.2920 (6)	0.10452 (11)	0.5520 (5)	0.0220 (9)	
N3	−0.0259 (6)	0.13077 (12)	0.3685 (5)	0.0214 (9)	
N4	0.1927 (7)	0.22538 (14)	0.2626 (7)	0.0364 (12)	
C1	0.5913 (9)	0.20210 (16)	0.6668 (8)	0.0344 (13)	
H1	0.5322	0.2252	0.6180	0.041*	
C2	0.7726 (9)	0.2048 (2)	0.7722 (8)	0.0450 (16)	
H2	0.8307	0.2295	0.8015	0.054*	
C3	0.8672 (9)	0.17072 (19)	0.8340 (8)	0.0415 (15)	
H3	0.9939	0.1719	0.9023	0.050*	
C4	0.7740 (9)	0.13467 (19)	0.7944 (8)	0.0323 (13)	
H4	0.8378	0.1112	0.8343	0.039*	
C5	0.5864 (9)	0.13370 (17)	0.6959 (7)	0.0232 (12)	
C6	0.4710 (8)	0.09759 (15)	0.6481 (6)	0.0232 (11)	
C7	0.5267 (8)	0.05923 (15)	0.6877 (7)	0.0293 (13)	
H7	0.6493	0.0536	0.7566	0.035*	
C8	0.3979 (8)	0.02930 (15)	0.6235 (7)	0.0308 (13)	
H8	0.4354	0.0031	0.6481	0.037*	
C9	0.2163 (7)	0.03682 (14)	0.5247 (7)	0.0282 (16)	
H9	0.1295	0.0161	0.4828	0.034*	
C10	0.1642 (6)	0.07567 (11)	0.4881 (12)	0.0219 (8)	
C11	−0.0190 (8)	0.09089 (14)	0.3876 (7)	0.0222 (11)	
C12	−0.1803 (8)	0.06726 (16)	0.3173 (7)	0.0285 (12)	
H12	−0.1745	0.0398	0.3300	0.034*	
C13	−0.3493 (8)	0.08484 (17)	0.2285 (7)	0.0302 (13)	
H13	−0.4597	0.0694	0.1793	0.036*	
C14	−0.3552 (9)	0.12527 (18)	0.2122 (8)	0.0266 (14)	
H14	−0.4706	0.1376	0.1535	0.032*	
C15	−0.1895 (8)	0.14736 (16)	0.2831 (8)	0.0269 (12)	
H15	−0.1927	0.1749	0.2705	0.032*	
O4	0.1900 (5)	0.09192 (10)	1.0047 (10)	0.0396 (10)	
O5	0.2336 (6)	0.03043 (12)	1.0689 (6)	0.0505 (11)	
O6	−0.0235 (6)	0.04770 (11)	0.8974 (5)	0.0425 (10)	
N5	0.1345 (5)	0.05686 (11)	0.9909 (9)	0.0287 (8)	

### Atomic displacement parameters (Å^2^)

**Table:**

	*U*^11^	*U*^22^	*U*^33^	*U*^12^	*U*^13^	*U*^23^
Pd1	0.02599 (16)	0.01582 (15)	0.02517 (16)	0.00131 (14)	−0.0033 (4)	−0.0007 (3)
O1	0.049 (2)	0.0166 (18)	0.037 (3)	0.0048 (15)	−0.0099 (16)	0.0013 (15)
O2	0.064 (3)	0.036 (2)	0.040 (2)	−0.010 (2)	−0.010 (2)	0.000 (2)
O3	0.059 (3)	0.020 (2)	0.083 (3)	−0.003 (2)	−0.020 (2)	0.021 (2)
N1	0.030 (2)	0.025 (3)	0.023 (2)	−0.0059 (18)	−0.0040 (19)	−0.0003 (19)
N2	0.0229 (19)	0.019 (2)	0.024 (2)	0.0028 (17)	0.0051 (17)	0.0003 (16)
N3	0.025 (2)	0.019 (2)	0.020 (2)	−0.0004 (17)	0.0018 (18)	−0.0021 (19)
N4	0.030 (2)	0.026 (3)	0.054 (3)	−0.007 (2)	−0.016 (2)	0.011 (3)
C1	0.042 (3)	0.024 (3)	0.037 (3)	−0.002 (3)	−0.004 (3)	0.000 (3)
C2	0.051 (4)	0.047 (4)	0.038 (3)	−0.021 (3)	−0.008 (3)	−0.002 (3)
C3	0.035 (3)	0.063 (4)	0.027 (3)	−0.014 (3)	−0.009 (3)	0.003 (3)
C4	0.029 (3)	0.044 (4)	0.025 (3)	0.003 (3)	0.001 (3)	0.006 (3)
C5	0.026 (3)	0.026 (3)	0.018 (3)	0.000 (2)	0.000 (2)	0.003 (2)
C6	0.025 (3)	0.027 (3)	0.018 (3)	0.003 (2)	0.003 (2)	0.005 (2)
C7	0.027 (3)	0.029 (3)	0.031 (3)	0.003 (2)	0.002 (2)	0.009 (3)
C8	0.034 (3)	0.020 (3)	0.039 (3)	0.008 (2)	0.007 (3)	0.012 (2)
C9	0.029 (2)	0.019 (2)	0.037 (5)	−0.0034 (19)	0.008 (2)	0.000 (2)
C10	0.0234 (19)	0.018 (2)	0.024 (2)	0.0004 (15)	0.000 (5)	−0.001 (4)
C11	0.026 (3)	0.018 (3)	0.022 (3)	0.002 (2)	0.006 (2)	0.000 (2)
C12	0.028 (3)	0.024 (3)	0.033 (3)	−0.006 (2)	0.007 (2)	−0.004 (2)
C13	0.024 (3)	0.037 (4)	0.030 (3)	−0.004 (2)	0.003 (2)	−0.009 (3)
C14	0.021 (3)	0.034 (4)	0.024 (4)	0.006 (3)	0.004 (3)	0.001 (3)
C15	0.029 (3)	0.024 (3)	0.027 (3)	0.007 (2)	0.003 (3)	−0.002 (2)
O4	0.0415 (17)	0.0291 (18)	0.048 (3)	−0.0045 (14)	−0.002 (3)	−0.003 (3)
O5	0.041 (2)	0.042 (3)	0.068 (3)	0.011 (2)	−0.020 (2)	0.013 (2)
O6	0.039 (2)	0.039 (2)	0.050 (2)	−0.0026 (18)	−0.0192 (19)	0.002 (2)
N5	0.0249 (17)	0.035 (2)	0.027 (2)	0.0011 (16)	0.004 (4)	−0.007 (4)

### Geometric parameters (Å, º)

**Table:**

Pd1—N2	1.917 (4)	C5—C6	1.466 (8)
Pd1—N1	2.026 (4)	C6—C7	1.381 (7)
Pd1—O1	2.028 (3)	C7—C8	1.380 (7)
Pd1—N3	2.030 (4)	C7—H7	0.9400
O1—N4	1.315 (6)	C8—C9	1.374 (7)
O2—N4	1.228 (6)	C8—H8	0.9400
O3—N4	1.218 (6)	C9—C10	1.386 (6)
N1—C1	1.332 (6)	C9—H9	0.9400
N1—C5	1.366 (7)	C10—C11	1.460 (7)
N2—C6	1.346 (6)	C11—C12	1.388 (7)
N2—C10	1.349 (6)	C12—C13	1.379 (7)
N3—C15	1.328 (6)	C12—H12	0.9400
N3—C11	1.363 (6)	C13—C14	1.379 (8)
C1—C2	1.379 (7)	C13—H13	0.9400
C1—H1	0.9400	C14—C15	1.381 (8)
C2—C3	1.379 (9)	C14—H14	0.9400
C2—H2	0.9400	C15—H15	0.9400
C3—C4	1.387 (8)	O4—N5	1.245 (5)
C3—H3	0.9400	O5—N5	1.236 (6)
C4—C5	1.380 (7)	O6—N5	1.245 (5)
C4—H4	0.9400		
			
N2—Pd1—N1	81.26 (17)	N2—C6—C7	119.1 (5)
N2—Pd1—O1	176.54 (15)	N2—C6—C5	112.9 (4)
N1—Pd1—O1	95.62 (15)	C7—C6—C5	128.0 (5)
N2—Pd1—N3	81.03 (16)	C8—C7—C6	118.4 (5)
N1—Pd1—N3	162.23 (16)	C8—C7—H7	120.8
O1—Pd1—N3	102.04 (15)	C6—C7—H7	120.8
N4—O1—Pd1	115.4 (3)	C9—C8—C7	121.8 (5)
C1—N1—C5	119.8 (5)	C9—C8—H8	119.1
C1—N1—Pd1	127.7 (4)	C7—C8—H8	119.1
C5—N1—Pd1	112.5 (4)	C8—C9—C10	118.4 (5)
C6—N2—C10	123.3 (4)	C8—C9—H9	120.8
C6—N2—Pd1	118.2 (3)	C10—C9—H9	120.8
C10—N2—Pd1	118.3 (3)	N2—C10—C9	118.9 (5)
C15—N3—C11	119.8 (4)	N2—C10—C11	112.6 (4)
C15—N3—Pd1	127.9 (3)	C9—C10—C11	128.4 (4)
C11—N3—Pd1	112.4 (3)	N3—C11—C12	120.8 (5)
O3—N4—O2	123.9 (5)	N3—C11—C10	115.5 (4)
O3—N4—O1	117.5 (5)	C12—C11—C10	123.7 (4)
O2—N4—O1	118.6 (4)	C13—C12—C11	118.9 (5)
N1—C1—C2	121.9 (6)	C13—C12—H12	120.6
N1—C1—H1	119.1	C11—C12—H12	120.6
C2—C1—H1	119.1	C12—C13—C14	119.6 (5)
C1—C2—C3	118.9 (6)	C12—C13—H13	120.2
C1—C2—H2	120.5	C14—C13—H13	120.2
C3—C2—H2	120.5	C13—C14—C15	119.1 (5)
C2—C3—C4	119.5 (5)	C13—C14—H14	120.4
C2—C3—H3	120.3	C15—C14—H14	120.4
C4—C3—H3	120.3	N3—C15—C14	121.8 (5)
C5—C4—C3	119.2 (6)	N3—C15—H15	119.1
C5—C4—H4	120.4	C14—C15—H15	119.1
C3—C4—H4	120.4	O5—N5—O4	121.2 (5)
N1—C5—C4	120.5 (5)	O5—N5—O6	118.5 (4)
N1—C5—C6	115.1 (5)	O4—N5—O6	120.3 (5)
C4—C5—C6	124.3 (5)		
			
Pd1—O1—N4—O3	−174.4 (3)	C6—C7—C8—C9	−0.9 (8)
Pd1—O1—N4—O2	5.6 (6)	C7—C8—C9—C10	0.6 (8)
C5—N1—C1—C2	−2.6 (8)	C6—N2—C10—C9	1.0 (9)
Pd1—N1—C1—C2	178.2 (4)	Pd1—N2—C10—C9	175.9 (5)
N1—C1—C2—C3	4.2 (9)	C6—N2—C10—C11	−179.7 (4)
C1—C2—C3—C4	−2.4 (9)	Pd1—N2—C10—C11	−4.8 (7)
C2—C3—C4—C5	−0.9 (8)	C8—C9—C10—N2	−0.6 (9)
C1—N1—C5—C4	−0.8 (8)	C8—C9—C10—C11	−179.7 (6)
Pd1—N1—C5—C4	178.5 (4)	C15—N3—C11—C12	0.5 (7)
C1—N1—C5—C6	−179.4 (5)	Pd1—N3—C11—C12	179.3 (4)
Pd1—N1—C5—C6	−0.2 (5)	C15—N3—C11—C10	−178.3 (5)
C3—C4—C5—N1	2.5 (8)	Pd1—N3—C11—C10	0.5 (6)
C3—C4—C5—C6	−179.0 (5)	N2—C10—C11—N3	2.6 (8)
C10—N2—C6—C7	−1.4 (8)	C9—C10—C11—N3	−178.2 (6)
Pd1—N2—C6—C7	−176.3 (4)	N2—C10—C11—C12	−176.1 (5)
C10—N2—C6—C5	177.7 (5)	C9—C10—C11—C12	3.0 (11)
Pd1—N2—C6—C5	2.8 (5)	N3—C11—C12—C13	−0.4 (8)
N1—C5—C6—N2	−1.6 (6)	C10—C11—C12—C13	178.3 (5)
C4—C5—C6—N2	179.8 (5)	C11—C12—C13—C14	−0.3 (8)
N1—C5—C6—C7	177.4 (5)	C12—C13—C14—C15	1.0 (8)
C4—C5—C6—C7	−1.2 (8)	C11—N3—C15—C14	0.1 (8)
N2—C6—C7—C8	1.3 (7)	Pd1—N3—C15—C14	−178.4 (4)
C5—C6—C7—C8	−177.7 (5)	C13—C14—C15—N3	−0.9 (9)

### Hydrogen-bond geometry (Å, º)

**Table:**

*D*—H···*A*	*D*—H	H···*A*	*D*···*A*	*D*—H···*A*
C3—H3···O2^i^	0.94	2.55	3.419 (7)	153
C4—H4···O6^ii^	0.94	2.37	3.303 (7)	172
C7—H7···O6^ii^	0.94	2.30	3.231 (6)	171
C8—H8···O5^iii^	0.94	2.43	3.088 (6)	127
C9—H9···O6^iv^	0.94	2.35	3.254 (6)	160
C13—H13···O5^v^	0.94	2.46	3.402 (7)	176
C15—H15···O3^vi^	0.94	2.38	3.280 (7)	161

Symmetry codes: (i) *x*+1, *y*, *z*+1; (ii) *x*+1, *y*, *z*; (iii) −*x*+1, −*y*, *z*−1/2; (iv) −*x*, −*y*, *z*−1/2; (v) *x*−1, *y*, *z*−1; (vi) *x*−1/2, −*y*+1/2, *z*.
